# Factors Related to Maternal Oral Health Status: Focus on Pregnant and Breastfeeding Women

**DOI:** 10.3390/healthcare9060708

**Published:** 2021-06-10

**Authors:** Eun Gyeong Kim, Sook Kyoung Park, Ju-Hee Nho

**Affiliations:** 1 Department of Nursing, Kunsan National University, Gunsan 54150, Korea; egkim@kunsan.ac.kr; 2 College of Nursing, Jeonbuk National University, Jeonju 54896, Korea; yoursky@jbnu.ac.kr

**Keywords:** community health survey, oral health status, pregnancy, breastfeeding

## Abstract

Oral health management is vital for pregnant women and their fetuses. This study analyzed the factors affecting maternal oral health status. It used secondary data obtained from the 2019 Korean Community Health Survey. Data were analyzed using SPSS 21.0 for complex descriptive statistics, independent t-test, Pearson’s correlation coefficients, and multiple regression analysis. Multiple regression analysis revealed that age (β = −0.01, *p* < *0*.001), maternal characteristics (β = −0.10, *p* < 0.001), education (β = −0.06, *p* = 014), subjective health status (β = 0.27, *p* < *0*.001, sleep duration (β = 0.07, *p* = *0*.003), breakfast frequency (β = −0.16, *p* < 0.001), unmet dental care needs (β = 0.35, *p* < 0.001), and depression (β = −0.02, *p* < 0.001) are key factors of maternal oral health status. Furthermore, oral health status is associated with mental health factors, such as depression. Therefore, the importance of maternal oral health among pregnant and breastfeeding women must be highlighted to promote the health of mothers and their babies.

## 1. Introduction

Women undergo many physical, mental, and emotional changes during pregnancy, due to which they experience fatigue, emotional sensitivity, and instability. Such changes can also affect the oral health of mothers [1]. The oral cavity not only protects the body from infection and helps in chewing and swallowing, but also plays a key role in psychosocial aspects, such as self-expression, communication, and beauty. Moreover, oral health is closely associated with systemic health [2]. Particularly, in pregnant women, changes in the endocrine system increase the bacteria in the mouth, causing the gums to bleed and swell. Additionally, consuming a variety of snacks during morning sickness increases the possibility of oral health problems. During pregnancy, women are more sensitive to stress and have lower resistance to infections, which leads to oral infections and oral health problems [3]. Studies have reported that the risk of dental caries increases during pregnancy because women frequently consume fermentable carbohydrates, such as sugar-rich drinks or meals, to prevent ketosis due to nausea and vomiting (hyperemesis gravidarum), thus leading to oral health problems [4]. Moreover, increased stress causes oral mucosa to become irritable because of decreased resistance to infection, and gestational gingivitis easily develops when infection or physical stimulation is received [4]. Pregnant women are more likely to develop periodontitis faster than the general population and have a higher risk of tooth loss than non-pregnant women [4]. Furthermore, changes in the environment of the oral cavity and hormonal changes weaken the walls of blood vessels, leading to swelling of the gums, increasing the vulnerability to periodontitis because such changes facilitate bacterial propagation due to the acidification of saliva [5].

However, many pregnant women do not undergo medical examinations despite oral health problems during pregnancy [3] because of socioeconomic and cultural factors, and myths, such as that dental examinations cannot be performed during pregnancy [6]. This can adversely affect the oral health of women who have experienced pregnancy and childbirth. It has also been reported that periodontitis in women during pregnancy increases the risk of preterm birth and infants with a low birth weight [7]. Furthermore, there are chances of the bacteria spreading to the children if a mother with untreated dental problems during pregnancy is careless during lactation [3]. In recent years, studies have focused on the relationship between oral health and breastfeeding, and their findings show that the duration of breastfeeding and the number of remaining natural teeth are inversely proportional among menopausal women [8].

Studies indicated that pregnancy and childbirth are associated with a higher number of missing teeth and that there is a higher edentulous rate among women than among men [9]. Among women in the childbearing age group (18–34 years), 33.6% aged 18–24 years, 41.1% aged 25–29 years, and 40.6% aged 30–34 years believed that they experienced problems related to their oral health [10]. Therefore, healthcare providers are interested in the oral health care management of women in the childbearing age group.

Gestational oral disease is related to the level of accumulation of plaque, oral health status, occupational level, education level, and the degree of oral care before pregnancy. There are various causes of gestational oral diseases, and their incidence is high [11]. Therefore, early detection and management of risk factors are crucial for ensuring the good oral health of pregnant women. For this, comprehensive and multifaceted efforts are required. Additionally, oral health management during pregnancy not only reduces the likelihood of developing or worsening oral diseases, but also facilitates thorough oral health management, active treatment, and prevention. The chances of maternal infection can be reduced by reducing the number of bacteria in the oral cavity [12]. Furthermore, a mother’s interest in oral health affects her child’s oral health before school; therefore, active efforts should be made to promote oral health management [13].

As discussed above, the acidification of saliva due to increased monosaccharide intake during pregnancy leads to various symptoms, such as nausea and vomiting [1]; an increase in estrogen and progesterone leads to the the vascular wall becoming weakened; and the risk of periodontal disease in pregnant women is high [14,15]. The oral health of pregnant women affects not only maternal systemic health, but also the birth outcome of the fetus. Therefore, the oral health management of pregnant women is important for the health of both the mother and fetus. 

However, only few studies in Korea focus on maternal oral health status, and most surveys are centered around specific groups [5,16]. Few studies have been conducted on a large number of factors related to oral health status in pregnant and lactating women. Therefore, data from a national-level community health survey were used in this study to determine methods for effectively managing maternal oral health based on the results of health examinations conducted to identify factors related to oral health status.

The purpose of this study is to understand the oral health status of pregnant and breastfeeding women in Korea, and to investigate the influencing factors of oral health status. The specific purpose is as follows.

First, to understand the oral health status of pregnant and breastfeeding women. Second, to identify relationships between oral health status and other variables (general characteristics and health behaviors). Third, to analyze the factors related to the oral health of pregnant and breastfeeding women.

## 2. Methods

### 2.1. Design 

This study conducted a secondary analysis to identify and analyze factors related to maternal oral health. 

### 2.2. Data Collection Method and Participants

This study used data from the 2019 Korean Community Health Survey (CHS). The CHS, launched in 2008, aims at establishing and evaluating local healthcare plans and producing comparable local health statistics. The questions used in the survey addressed health-relating behaviors, such as smoking, alcohol consumption, physical activity, brushing, breakfast, nutritional labeling and diet, chronic disease morbidity and treatment level, weight control behavior and obesity level, and wearing seat belts. The survey was based on 128 health indicators, such as safety awareness, oral health, mental health, and quality of life index. Surveys of up to 281 questions (approximately 201 for the whole country, 80 for selected regions) and measurement surveys were conducted through household visits (approximately 230,000 total samples, an average of 900 people per region). The 2019 CHS was administered to all residents over the age of 19 residing in each region. It selected sample points through probability proportional system extraction, such that an average of 900 samples could be surveyed at 253 municipal health centers across the country. The surveyed households were chosen according to the systematic extraction method at the sample points wherein adults over 19 years of age were targeted and selected. A total of 229,099 people participated in the 2019 CHS. In our study, data from 1630 women who answered “pregnant” or “breastfeeding after childbirth” to the question, “Are you currently menstruating?” among women aged 19 to 55 were used in the final analysis.

A total of 229,099 people participated in the 2019 CHS.

An investigator trained in the 1:1 interview (computer-assisted personal interviewing, CAPI) method collected raw data using a laptop equipped with a survey program between August 16 and October 31, 2019. This study used original data from which logical errors and outliers were removed and personal identification information was deleted.

### 2.3. Measurement

#### 2.3.1. General Characteristics

The general characteristics included age, maternal characteristics, and education level. Maternal characteristics were classified as either “pregnancy” or “breastfeeding”. Education levels in the CHS were classified into “no education”, “elementary school”, “junior high school”, “high school”, “2/3-year college”, “4-year college”, and “graduate school or higher”. However, in this study, the higher education levels were reclassified as “less than 4-year university” and “4-year university or higher”. 

#### 2.3.2. Oral Health Status 

Participants’ oral health status was defined as an answer on the five-point Likert scale (1 = “very bad”, 2 = “poor”, 3 = “moderate”, 4 = “good”, and 5 = “very good”) to the item asking, “When you think about yourself, how do you feel about your oral health, including teeth and gums?”, and higher scores indicated good oral health. 

#### 2.3.3. Health Behaviors

The health behaviors of participants were measured based on various factors, such as subjective health status, depression, stress, breakfast frequency, sleep duration, and unmet dental care needs. Subjective health status was measured on a five-point Likert scale (1 = “very bad”, 2 = “bad”, 3 = “fair”, 4 = “good”, and 5 = “very good”), and higher scores indicated good health status. For depression, the Korean version of the Patient Health Questionnaire-9 (PHQ-9) depression screening tool was used. At the time of development, the PHQ-9′s internal consistency reliability (Cronbach’s alpha) was 0.84. In this study, the Cronbach’s alpha was 0.77. Stress was measured using a four-point Likert scale (1 = “I hardly feel stress”, 2 = “I feel a little stress”, 3 = “I feel a lot of stress”, and 4 = “I feel very much stress”), with higher scores indicating higher stress levels. Breakfast frequency was defined as an answer on the 4-point Likert scale (1 = “5–7 times/week”, 2 = “3–4 times/week”, 3 = “1–2 times/week”, 4 = “0 times/week”) to the item asking, “How many times a week did you eat breakfast in the past year?”. In this study, breakfast frequency was reclassified as “more than 5 times a week” and “less than 5 times a week”. Sleep duration was classified into “more than 8 h” and “less than 8 h”. Unmet dental care needs were determined based on whether participants responded with “Yes” to the question, “Did you believe that you required dental treatment over the last year but have continued to not receive medical treatment?”.

### 2.4. Ethical Considerations and Statistical Analysis

For ethical consideration of the participants, the raw data were requested from the KCHS homepage (http://chs.cdc.go.kr/, accessed on 25 March 2021), and were obtained with all private information remaining anonymous. This study is a secondary analysis of the data of those who participated in the community health survey. The original data were requested according to the original data use and related statistical data users’ compliance statements and data use plans. This was followed by approval on the website of the CHS, and raw data excluding personal identification information were provided. Raw data of the community health survey refers to data that are disclosed to the public after removing inappropriate answers to questions (e.g., answers outside of the categories presented in the questions) from the original data, converting personal information to private information according to the Personal Information Protection Act that has been enforced since 2011, and deleting personal identification information. Thus, in this study, participant anonymity was guaranteed because raw data classified by virtual numbers that did not contain personal identification information were provided.

Data analysis was performed using IBM SPSS Statistics 21.0. A complex independent t-test and chi-square test were performed to determine the difference in oral health status based on participants’ characteristics. Complex multiple regression analysis was conducted to confirm the factors related to the oral health status of pregnant and lactating women. According to the CHS data, which are a composite sample design, individual weights were applied to estimate the population. Cronbach’s α coefficient was calculated to verify the reliability of the tool.

## 3. Results

### 3.1. General Characteristics of Participants

A total of 1630 participants were examined. Table 1 presents the results of analyzing the general characteristics and oral health status and health behaviors of these participants. The mean age of participants was 33.5 years, and 65.8% of the participants were pregnant women. Regarding the level of education, 56.5% fell under the “4-year university or higher” category. The mean score of oral health status was 3.09. The mean score of subjective health status was 3.54, the mean depression score was 11.48, and the mean stress score was 2.04. In the breakfast frequency, less than 5 times per week accounted for 50.3%. Regarding the sleeping duration, 60.7% experienced less than 8 h of sleep, and 76.7% responded with “No” to the question regarding unmet dental care needs (Table 1). 

### 3.2. Relationships between Oral Health Status and Variables of Participants

The results of the correlation analysis showed that age (r = −0.05, *p* = 0.032), depression (r = −0.20, *p* < 0.001), and stress (r = −0.13, *p* < 0.001) were significantly negatively correlated to oral health status. A significant positive correlation was revealed between oral health status and subjective health status (r = 0.28, *p* < 0.001). Oral health status was significantly higher among pregnant participants (t = −4.82, *p* < 0.001), those who graduated from a 4-year university or higher (t = −2.90, *p* = 0.004), those who ate breakfast ≥ “5 days or more” (t = −7.80, *p* < 0.001), and those who responded with “No” to the question regarding unmet dental care needs (t = 14.51, *p* < 0.001) (Table 2). 

### 3.3. Analysis of Factors Related to Oral Health Status

A multiple regression model was used to analyze factors related to oral health status (Table 3). In terms of general characteristics, as the age decreased (β = −0.01, *p* < 0.001), in the case of pregnant women (β = −0.10, *p* < 0.001), and in those who had graduated from 4-year universities or higher (β = −0.06, *p* = 014), the oral health status score was increased significantly. In health behaviors, as the subjective health score increased (β = 0.27, *p* < 0.001), in those who experienced sleep duration of “8 h or more” (β = 0.07, *p* = 0.003), and in those who responded with “no” to the unmet dental care needs question (β = 0.35, *p* < 0.001), the oral health status score was increased. In addition, as the depression and stress score increased (β = −0.02, *p* < 0.001), in those who ate breakfast “5 days or more” (β = −0.16, *p* < 0.001), the oral health status score was significantly decreased. The explanatory power of these variables for explaining oral health status was 17.6%, and the model was considered suitable (Wald F = 80.23, *p* < 0.001) (Table 3). 

## 4. Discussion

Environmental creation and oral health management during pregnancy and breastfeeding, which can prevent the most detrimental factors for oral health in women’s life cycles, are important. Therefore, this study aimed to prepare basic data for maternal oral health promotion programs by identifying factors related to oral health status with respect to pregnancy and breastfeeding. 

Lower maternal ages, pregnancy, and education levels of four-year college or higher were associated with better oral health status. This supports the findings of previous studies showing that the lower the education level, the worse the oral health [17,18,19]. Recently, as the average age of first-time mothers has increased, oral health management has become more important. Therefore, policy support is needed to implement stronger measures to improve oral health management and services for pregnant women with low education levels.

In lactating women, lactation is associated with a change in maternal calcium enhancement [20], and breastfeeding has been reported to cause maternal water loss and oral health problems [21]. Therefore, healthcare providers should try to discourage dehydration, which can be aided by educational intervention. 

A better subjective health status indicated better oral health status. Higher depression scores indicated worse oral health status. Studies have noted a significant correlation between depression and periodontal disease among pregnant women; lower depression scores indicated lower incidence of periodontal disease [22]. According to previous research, mental health problems, such as stress and depression, are related to the occurrence of periodontal disease. Stress causes various physical changes by directly altering the body’s immune system. Changes in the immune system that are caused by stress are related not only to the occurrence of periodontal diseases, such as acute gingivitis and acute periodontitis, but also to the occurrence of heart disease and diabetes. The secretion of cortisol due to stress is transmitted to the fetus through the placenta and can cause neurological problems in the fetus [23,24]. In particular, the mental health problems of pregnant women have been proven to be related to adverse health effects in the child, such as low birth weight, and these also affect the child’s mental behavior after birth [25]. Depression in pregnant women can lead to negative consequences, such as premature birth [26]. Therefore, it is necessary to actively screen and manage stress and depression to improve fetal and maternal health. Additionally, since many women experience depression after childbirth, it is necessary to establish a social and family support system to address this problem.

Among those who had a sleeping duration of less than eight hours and experienced unmet dental care needs, oral health was found to be poor. Studies have shown that inadequate sleep duration leads to changes in the saliva and oral environment, thereby causing oral diseases, such as periodontal disease and dental caries, and those who sleep less than six hours have a high risk of periodontal disease [27]. Studies have reported that sleep patterns change due to various physiological and physical changes during pregnancy, and various problems that interfere with sleep are common [28]. Kempler et al. [29] showed that the quality of sleep improved and depressive symptoms decreased through education during pregnancy. Therefore, the importance of sleep should be considered in the oral health care program for pregnant women. Moreover, in the case of unmet dental care needs, the experience rate is higher among women than among men [30]. Thus, support for unmet dental care needs for pregnant women is required.

This study has several limitations. There is a limit to revealing specific influencing factors because they do not specifically reflect maternity-related characteristics. This is because the data were taken from a self-reported study that was evaluated by the participants themselves, and the analysis items were simplified. Moreover, it is impossible to explain the causal relationship using a cross-sectional study design. However, it is considered a meaningful study because it identifies factors related to oral health, considering that studies focusing on oral health among pregnant women are insufficient.

A cohort study is required to focus on the relationship between maternal breastfeeding and oral health status during pregnancy through follow-up. Furthermore, socio-cultural factors and oral health beliefs have been reported as impeding factors for oral health management in pregnant and lactating women. Therefore, further studies are required to confirm these claims.

## 5. Conclusions

This study investigated the relationship between the characteristics and health status of pregnant women and their oral health status. The results showed that age, education, subjective health status, depression, sleep time, and unmet dental care were related to maternal oral health status. 

The oral health status of pregnant and lactating women is related to depression. Therefore, an oral health behavior promotion program that considers psychological problems must be developed. A management strategy must be established for the practice of healthy behaviors, such as ensuring sufficient sleep. Based on the results of this study, a systematic survey should be conducted periodically to fully understand maternal oral health status at the national level. Standardization of educational programs and guidelines should be developed to enable comprehensive oral health education.

## Figures and Tables

**Table 1:** General characteristics, oral health status, and health behaviors of participants (N = 1630).

Variables	Categories	Weighted % or Mean ± SE (min-max)
General characteristics		
Age (years)		33.5 ± 0.1 (19–55)
Maternal characteristics	Pregnant	65.8
	Breast feeding	34.2
Education	≥University	56.5
	<University	43.5
Oral health status		3.09 ± 0.01(1–5)
Health behaviors		
Subjective health status		3.54 ± 0.01 (1–5)
Depression		11.48 ± 0.05 (9–32)
Stress		2.04 ± 0.01 (1–4)
Breakfast frequency (day/week)	≥5	49.7
	<5	50.3
Sleep duration (hour/day)	≥8	39.3
	<8	60.7
Unmet dental care needs	Yes	23.3
	No	76.7

SE: standard error.

**Table 2:** Relationships between oral health status and variables of participants (*N* = 1630).

Variables	Categories	Oral Health Status (Mean ± SE)	t or r	*p*
General characteristics				
Age (years)			−0.05	0.032
Maternal characteristics	Pregnant	3.13 ± 0.01	−4.82	<0.001
	Breastfeeding	3.01 ± 0.02		
Education	≥University	3.12 ± 0.02	−2.91	<0.004
	<University	3.05 ± 0.02		
Health behaviors				
Subjective health status			0.28	<0.001
Depression			−0.20	<0.001
Stress			−0.13	<0.001
Breakfast frequency (day/week)	≥5	3.18 ± 0.02	−7.80	<0.001
	<5	3.00 ± 0.02		
Sleep duration (hour/day)	≥8	3.11 ± 0.02	−1.47	0.142
	<8	3.08 ± 0.02		
Unmet dental care needs	Yes	3.02 ± 0.03	14.51	<0.001
	No	3.19 ± 0.04		

SE: standard error.

**Table 3:** The effect factor of oral health status.

Variable		β	SE	t	*p*
Constant		2.51	0.132	19.016	<0.001
General characteristics	Age	−0.01	0.002	−4.10	<0.001
	Maternal characteristics (breastfeeding)	−0.10	0.023	−4.33	<0.001
	Education (<university)	−0.06	0.024	−2.49	0.014
Health behavior	Subjective health status	0.27	0.017	15.68	<0.001
	Depression	−0.02	0.004	−5.90	<0.001
	Stress	−0.02	0.017	−0.90	0.370
	Breakfast frequency (<5)	−0.16	0.024	−6.48	<0.001
	Sleep duration (≥8)	0.07	0.025	2.98	0.003
	Unmet dental care needs (no)	0.35	0.034	10.29	<0.001
R^2^ = 0.176, Wald F = 80.23, *p* <0.001					

SE: standard error.

## Data Availability

The data presented in this study are available on request from the corresponding author.
